# Mitochondrial Stress Responses and “Mito-Inflammation” in Cystic Fibrosis

**DOI:** 10.3389/fphar.2020.581114

**Published:** 2020-09-30

**Authors:** Simone Patergnani, Veronica A.M. Vitto, Paolo Pinton, Alessandro Rimessi

**Affiliations:** ^1^ Department of Medical Sciences and Laboratory for Technologies of Advanced Therapies (LTTA), University of Ferrara, Ferrara, Italy; ^2^ Center of Research for Innovative Therapies in Cystic Fibrosis, University of Ferrara, Ferrara, Italy

**Keywords:** mitochondria, inflammation, cystic fibrosis, mitochondrial stress response, lung

## Abstract

Cystic fibrosis (CF) is a genetic disease associated to mutations in the cystic fibrosis transmembrane conductance regulator gene, which results in the alteration of biological fluid and electrolyte homeostasis. The characteristic pathological manifestation is represented by exaggerated proinflammatory response in lung of CF patients, driven by recurrent infections and worsen by hypersecretion of proinflammatory mediators and progressive tissue destruction. Treating inflammation remains a priority in CF. However, current anti-inflammatory treatments, including non-steroidal agents, are poorly effective and present dramatic side effects in CF patients. Different studies suggest an intimate relationship between mitochondria and CF lung disease, supporting the hypothesis that a decline in mitochondrial function endorses the development of the hyperinflammatory phenotype observed in CF lung. This allowed the implementation of a new concept: the “mito-inflammation,” a compartmentalization of inflammatory process, related to the role of mitochondria in engage and sustain the inflammatory responses, resulting a druggable target to counteract the amplification of inflammatory signals in CF. Here, we will offer an overview of the contribution of mitochondria in the pathogenesis of CF lung disease, delving into mitochondrial quality control responses, which concur significantly to exacerbation of CF lung inflammatory responses. Finally, we will discuss the new therapeutic avenues that aim to target the mito-inflammation, an alternative therapeutic advantage for mitochondrial quality control that improves CF patient’s inflammatory state.

## Introduction

Cystic fibrosis (CF) is a genetic and multi-organ disease, caused by mutations in the cystic fibrosis transmembrane conductance regulator (CFTR) gene, resulting in lacked or reduced expression and function of protein (Elborn, 2016). ΔF508 is the most common mutation that displays a frequency of ∼66% worldwide, which determine the inadequate processing of protein with consequent trapping in the endoplasmic reticulum (ER) (Marson et al., 2016). CFTR protein functions as cyclic adenosine monophosphate-regulated chloride channel and regulator of epithelial transport proteins, including epithelial sodium channel (Csanady et al., 2019). Defective CFTR alters fluid and electrolyte homeostasis, resulting in abnormally viscous and sticky secretions that at pulmonary level facilitates the adhesion and proliferation of pathogens, especially by *Pseudomonas aeruginosa* (*P. aeruginosa*), triggering exaggerated pro-inflammatory responses characterized by elevated secretion of pro-inflammatory mediators (such as the interleukin (IL)-8, IL-1β, and IL-18) (Malhotra et al., 2019). The intrinsic defects associated with CFTR deficiency and the repeated pulmonary infections induce hyperinflammation, leading to reduced bacterial clearance capacity and tissue damage in airways, compromising the respiration (Rafeeq and Murad, 2017).

While the underlying mechanisms that promote CF lung disease progression are not fully delineated, more studies highlighted the connection between mitochondrial health and CF pathogenesis, shading light on this organelle’s role in regulating host response and inflammation.

Mitochondria are central signaling hub that communicate with the cell to regulate several functions, such as metabolism, regulation of calcium (Ca^2+^) homeostasis, inflammation, stress responses, and cell death. Differently from other organelles, mitochondria present two membranes constituted by low levels of sphingolipids and cholesterol with exclusively components, such as posphosphatidylglycerol and cardiolipin (CL). The outer mitochondrial membrane (OMM) fully surrounds the inner mitochondrial membrane, separating the intermembrane space (IMS) from the matrix (Mejia and Hatch, 2016). They create a dynamic tubular and organized network, where the shape is continuously controlled by opposing fission and fusion events. These dynamic events are relevant during cell cycle in mitochondrial movement and in the interaction with other intracellular organelles (Nunnari and Suomalainen, 2012).

The interorganelle communication plays a key role in the mitochondrial functions, in particular with ER. Approximately, 5–20% of mitochondria surface is juxtaposed to specialized ER regions, linked by specific physical tethers at distance of 10–30 nm distance. Functional ER-mitochondria tethering is due by inositol 1,4,5-trisphosphate (IP3) receptors (IP3Rs), glucose-regulated protein 75, and voltage-dependent anion channels (VDAC) to transfer Ca^2+^ from ER to mitochondria (Giorgi et al., 2018a). While among structural tethering: the ER-located mitofusin 2 (MFN2) that builds a bridge with a heterodimer complex with MFN1/2 on OMM, and the integral ER protein vesicle-associated membrane protein-associated protein B (VAPB), which interacts with protein tyrosine phosphatase-interacting protein-51 (PTPIP51) (Lee and Min, 2018). Disruption or remodeling of ER-mitochondria tethering lead to a mismatch between the two organelles, causing dysregulation of lipid trafficking, Ca^2+^ signaling, autophagosome formation and apoptosis progression (Rowland and Voeltz, 2012; Rimessi et al., 2020a).

The mitochondria’s innermost compartment is the matrix, where resides the mitochondrial DNA (mtDNA) that contains genetic coding information for 13 proteins, core constituents of the mitochondrial respiratory complexes I-IV. The matrix is also site of replication and transcription of mtDNA, protein biosynthesis, and ATP synthesis. During this last process, electrons move along the respiratory complexes of the mitochondrial electron transport chain (mETC). This flow is coupled to the pumping of protons from matrix into IMS, creating an electromotive force used to produce ATP (Turrens, 2003). During this process, some electrons leak out of ETC, interact with oxygen to produce reactive oxygen species (ROS), in particular the superoxide anion (O_2_^-^). Complex-I (NADH dehydrogenase) and complex-III (CoQH2-cytochrome c reductase) result the primary sites where electron leak occurs and ROS are produced.

The mitochondrial membrane potential (Δψ) is the driving force necessary for protein import *via* translocase of outer membrane/translocase of inner membrane complex and for mitochondrial Ca^2+^-uptake into the matrix through the mitochondrial Ca^2+^ uniporter (MCU). The Ca^2+^-released from ER, *via* IP3Rs, may enter into matrix through VDAC and MCU (Giorgi et al., 2018b). Mitochondrial Ca^2+^ may stimulate OXPHOS, but upon cell stress, excessive mitochondrial Ca^2+^ accumulation induces ROS production, autophagic flux reduction, and mitochondrial permeability transition pore (mPTP) opening, which lead to an irreversible collapse of Δψ, swelling of mitochondria, and release of pro-apoptotic factors, such as cytochrome c (Pedriali et al., 2017).

In this review, we highlight the role of mitochondria in the pathogenesis of CF lung disease. In particular, we will discuss of role of mitochondrial quality control responses, which are modulated by defective CFTR and persistent infections, contributing significantly to CF lung hyperinflammation. The complete understanding of these alterations, their molecular mechanisms, and the importance of each compensatory pathways engaged will help us to find new CF therapy strategies.

## Mitochondrial Dysfunction in Cystic Fibrosis

Endogenous and/or environmental stresses may perturb the mitochondrial homeostasis and interfere with the steady-state activity of mitochondrial functions, promoting a state of mitochondrial stress characterized by the inability to maintain basal Δψ and mitochondrial ROS production, with repercussions on mitochondrial protein import, mitochondrial Ca^2+^ signaling, and oxidation state. All this could harm airway epithelial and immune cells, contributing to the development and exacerbation of CF lung disease. Studies revealed that the mitochondrial impairments in the CF lung and the mitochondrial quality control responses are associated with CFTR deficiency and inflammatory environment.

## Mitochondrial Defects Associated to CFTR Deficiency

The first evidence of mitochondrial defects related to CFTR deficiency was obtained in the 80s, showing that the oxygen consumption rate of isolated mitochondria from CF patients was affected due to complex-I and Na^+^/K^+^ ATPase alterations (Feigal and Shapiro, 1979; Shapiro et al., 1979). Consistently, the gene MT-ND4 and CISD1, fundamentals for a proper functioning of mETC, resulted downregulated in CF patient-derived tracheal cells (Valdivieso et al., 2007). Indeed, case reports of CF patients reported impairments in cytochrome c oxidase and in 6-phosphate dehydrogenase (Congdon et al., 1981; Battino et al., 1986). Oxygen consumption, complex-I activity, Δψ, and OXPHOS were also found dysregulated in CFTR-silencing intestinal epithelial and in F508del-CFTR airway cells (Kleme et al., 2018). However, treatment with “CFTR correctors,” including VX809 and 4,6,4’-trimethylangelicin, improved all the mitochondrial parameters, indicating that the CFTR rescue is linked to recovery of mitochondrial function (Atlante et al., 2016).

Other mitochondrial defects, such as the mitochondrial protein pattern, the intracellular pH and mitochondrial Ca^2+^ signaling have been described in CF to be sufficient to promote ROS production and membrane lipid peroxidation (Turrens et al., 1982; Picci et al., 1991; de Meer et al., 1995; Rimessi et al., 2015). Mitochondrial alteration due to oxidative stress has been also reported in CFTR-knockout mice, where oxidative mtDNA damage and reduced aconitase activity were described (Velsor et al., 2006). Also the ROS detoxification capacity in CF appears compromised. Low levels of mitochondrial reduced glutathione (mtGSH) and defects in GSH transport have been found in CF patient-derived airway cells and in CFTR-knockout, resulting in an altered extracellular ratio between reduced and oxidized GSH (Gao et al., 1999; Velsor et al., 2006; Kelly-Aubert et al., 2011). Accordingly, functional CFTR reintroduction restored mtGSH levels, attenuating the Δψ depolarization and IL-8 secretion^28^.

## Mitochondrial Defects Associated to Persistence Infections

Pathogens affect mitochondria, generally causing Δψ loss and mitochondrial fragmentation, to influence their intracellular survival or to evade host immunity (Tiku et al., 2020). Airway epithelial and immune cells are sensitive to pathogen upon infection, activating mitochondrial stress responses to preserve the mitochondrial homeostasis.

In uninfected conditions, no significant differences in mitochondrial physiology and in inflammatory profile were detected between human CF and non-CF airway cells. Both mitochondrial networks exhibited the classical ultrastructure integrity of a well-defined OMM with numerous pleomorphic cristae. Contrariwise, the exposition to *P. aeruginosa* strains displayed extensive mitochondrial swollen and fragmentation with derangement of cristae in CF airway epithelial cells, resulting in Δψ loss, excessive O_2_^-^ production, and nod-like receptor 3 (NLRP3) inflammasome activation (Rimessi et al., 2015). Using non-motile *P. aeruginosa* mutants, it has been demonstrated that the mitochondrial dysfunction in CF airway epithelial cells is triggered by the bacterial constituent flagellin through a Toll-like receptor 5 (TLR5)-dependent pathway (Rimessi et al., 2015). The recovery of mitochondrial integrity in CF airway epithelial cells during infection was obtained silencing or pharmacologically inhibiting MCU, with KB-R7943, indicating that mitochondrial Ca^2+^ signaling has a role in *P. aeruginosa*-dependent mitochondrial impairments in CF (Rimessi et al., 2015; Rimessi et al., 2020b).

These data show that a functional CFTR channel may prevent the *P. aeruginosa*-triggered mitochondrial dysfunction, regulating the susceptibility of airway cells to infection and thus conditioning the degree of innate immune response.

## Mitochondrial Stress Responses in Cystic Fibrosis

It is not yet entirely clear whether mitochondrial dysfunction is a trigger for, a consequence, or both for CF lung disease. In resting conditions, compensatory mitochondrial stress responses are transiently activated to restore the mitochondrial homeostasis, while during the recurrent infections, the chronic mitochondrial stress condition leads to amplification and persistence of these responses. Functional fusion complementation, mitophagy, mitochondrial unfolded protein response (UPR^mt^), and apoptosis are recruited to recover and preserve the mitochondrial homeostasis to regulate metabolism and innate immune response and cell viability. In CF, the persistent infections and the defects associated with CFTR deficiency alter the mitochondria quality control machinery, acquiring potential relevance to the disease state.

## Functional Fusion Complementation and Mitophagy

**Figure 1:**
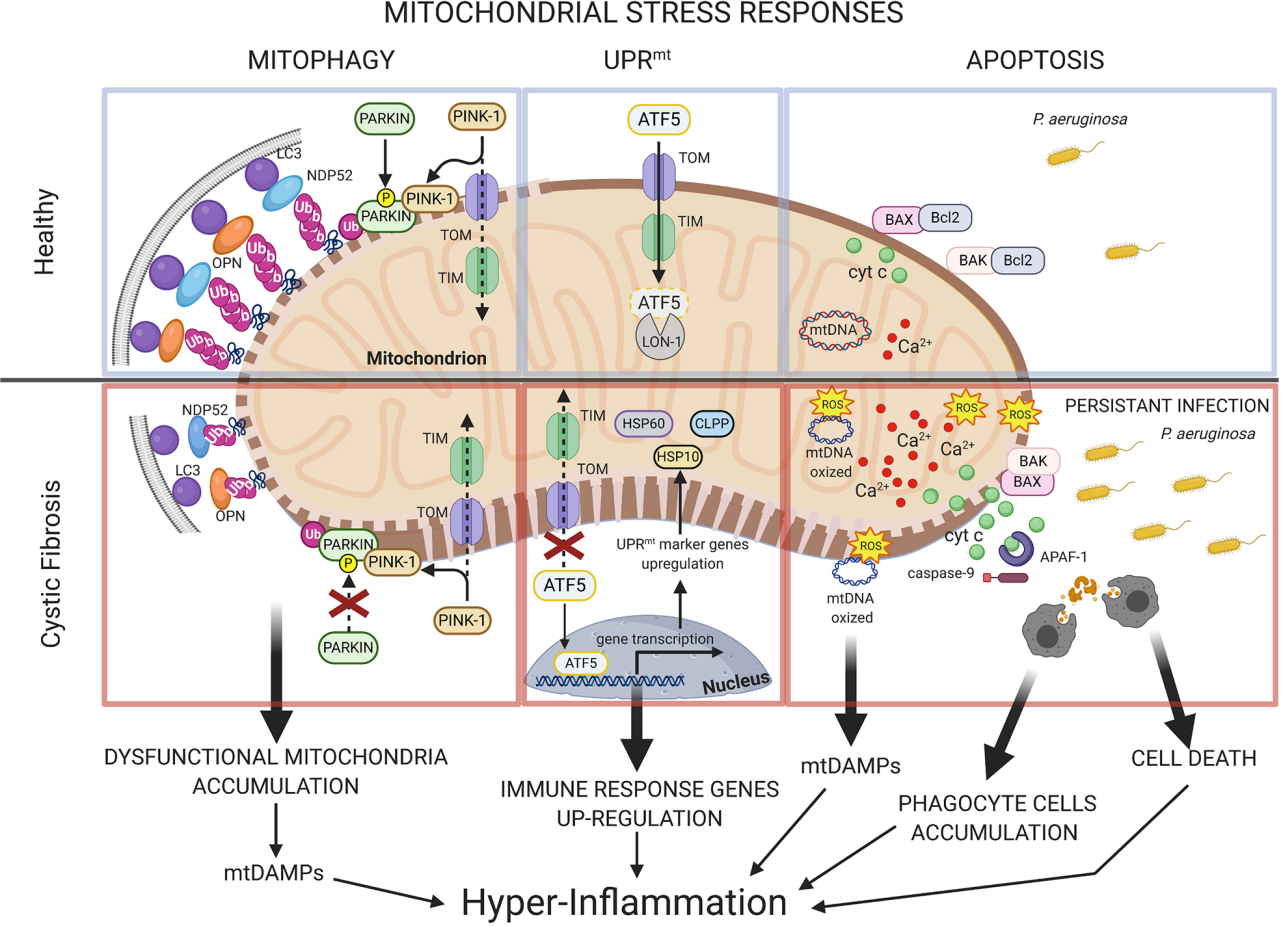
Mitochondrial stress responses in cystic fibrosis. Schematic representation of mitochondrial stress response pathways in healthy and in CF. In healthy condition, compensatory mitochondrial stress responses are transiently triggered to restore the mitochondrial homeostasis during *P. aeruginosa* infection. Damaged mitochondrial portions are removed by mitophagy, where the PINK1-Parkin signaling pathway promotes ubiquitination of OMM proteins while the mitophagic receptors, optineurin (OPN) and NDP52, act as adaptors to recruit autophagosomal membranes to mitochondria, interacting with LC3. In CF, defective mitophagy leads to dysfunctional mitochondria accumulation with consequent release of mitochondrial DAMPs, which contribute to hyper-inflammatory responses in CF airway cells during the persistent *P. aeruginosa* infection. The mammalian UPR^mt^ is regulated by the transcription factors ATF5, which in healthy condition is imported into mitochondria to be degraded. In CF, the persistent mitochondrial stress in airway cells induces abnormal UPR^mt^ activation with consequent nuclear translocation of ATF5, which supports the regulation of innate immunity response during pathogen infection. At front of the mitochondrial Ca^2+^-overload, a higher susceptibility to irreversible damages in response to pathogen infection occur in CF airway cells, contributing to promote organelle dysfunction and cell death. In CF, the exacerbation of inflammatory environment is due also by mtDAMPs release and by accumulation of phagocyte cells that intervene to scavenge the dying cells and pathogens. PTEN induced kinase 1 (PINK-1); Microtubule-associated proteins 1A/1B light chain 3B (LC3); Nuclear domain 10 protein 52 (NDP52); Optineurin (OPN); Translocase of the outer membrane (TOM); Translocase of the inner membrane (TIM); Activating Transcription Factor 5 (ATF5); Lon protease homolog 1 (LON-1); heat shock protein 60 (HSP60); heat shock protein 10 (HSP10); Caseinolytic Mitochondrial Matrix Peptidase Proteolytic Subunit (CLPP); mitochondrial deoxyribonucleic acid (mtDNA); cytochrome c (cyt c); B-cell lymphoma 2 protein (Bcl2); BCL2 Associated X protein (BAX); Bcl-2 homologous antagonist/killer protein (BAK); reactive oxygen species (ROS); calcium (Ca^2+^); *Pseudomonas aeruginosa* (*P. aeruginosa*); apoptotic protease activating factor-1 (APAF-1). This figure has been created with “BioRender.com”.

The morphology of mitochondrial network is regulated by fusion/fission events and mitophagy to sustain an adequate supply of healthy mitochondria. Mitochondrial fusion compensation optimize the functional efficiency of organelle under stressful conditions, allowing the exchange of materials among partially damaged mitochondria (Campello and Scorrano, 2010). The MFN1/2 are pivotal mediators of this process (Song et al., 2009). A sustained stress condition favors the segregation of damaged/depolarized parts of mitochondria in autophagosomes that then will be eliminated through lysosomal degradation by mitophagy. This catabolic process minimizes the quote of dysfunctional mitochondria, removing excess of ROS, oxidized mtDNA, and other mitochondrial dangerous factors relevant to disease state (Eisner et al., 2018) (**Figure 1**, mitophagy). Mitochondrial-targeted kinase PINK1 and E3 ubiquitin ligase Parkin have a central role for mitophagy. Parkin is recruited to OMM by perturbed mitochondrial import of PINK1 in stressed mitochondria, where catalyzes the ubiquitination of MFNs and other OMM proteins to sequester the organelle in autophagosome. At the same time, PINK1 contributes to strengthen the mitophagic response, phosphorylating both parkin and ubiquitin and recruiting the mitophagic receptors NDP-52 and optineurin to mitochondria (Koyano et al., 2014; Lazarou et al., 2015).

In airway epithelial cells the accumulation of NDP-52, optineurin and autophagic form of microtubule-associated protein light chain 3 (LC3-II) to mitochondria triggers mitophagy during *P. aeruginosa* infection. The minor mitochondrial redistribution of LC3-II indicated a slower kinetic of mitochondrial sequestration into autophagosome in CF cells respect to non-CF cells, confirmed also by a reduced recruitment of Parkin to stressed mitochondria (Rimessi et al., 2020b) (**Figure 1**, mitophagy). Defective mitophagy led to excessive O_2_^-^ production and NLRP3 inflammasome activation in CF airway cells during *P. aeruginosa* infection (Rimessi et al., 2015; Rimessi et al., 2020b). Similar defect was emerged also in xenophagy, the selective autophagic response involved to sequester into the cell invading pathogens (Gatica et al., 2018). The higher number of colony-forming unit/ml and of interactions between xenophagic receptors and invading *P. aeruginosa* in CF airway cells compared with non-CF cells, indicated a reduced bacterial clearance capacity of CF airway cells, resulting in further cell stress and pyroptosis induction (Rimessi et al., 2020b). The worsening of mitophagy and xenophagy in CF cells during infection is consequence of an enhanced ER-mitochondria juxtaposition, which making the organelles more prone to interorganelle Ca^2+^-exchange. CF airway cells exposed to *P. aeruginosa* showed increased expression and interaction between ER protein VAPB and OMM protein PTPIP51, which induced tightening of tethers and concomitant impairment of selective autophagic responses (Rimessi et al., 2020b). Pharmacologically control of mitochondria Ca^2+^-uptake, by MCU inhibitor KB-R7943, abrogated the inhibitory effects of VAPB and PTPIP51 on the selective autophagic responses, promoting cellular and mitochondrial resistance to infection and inflammatory reduction (Rimessi et al., 2020b). This highlights a central role for mitochondrial stress in progression of CF lung inflammatory state, with detrimental repercussions on the autophagic responses, which may further affect the expression, trafficking, and function of CFTR channel (Luciani et al., 2010; Villella et al., 2013). Defective macroautophagy in CF has been also associated to upregulation of transglutaminase (TG2), which led to ROS production and decrease of aggresomes clearance (Luciani et al., 2010). The rescue of autophagy, mediating antioxidants, cystamine (TG2 inhibitor), or modulators of Ca^2+^-dependent signaling (KB-R7943), resulted in improved CFTR transport to PM, reduced oxidative stress, and cytokines release in CF airway cells^30,37^.

## Mitochondrial Unfolded Protein Response

Perturbed mitochondrial protein import and the accumulation misfolded proteins within of organelle induces UPR^mt^ activation, a transcriptional program that results in a number of cellular- and mito-protective outcomes (Melber and Haynes, 2018). In *C. elegans*, the *P. aeruginosa* exposure induced mitochondrial stress and recruitment of stress-activated transcription factor 1, which induced the transcription of mitochondrial chaperones [such as heat shock protein (HSP) 10 and HSP 60], proteases (including the caseinolytic mitochondrial matrix peptidase proteolytic subunit), ROS detoxification, and innate immune genes, resulting a key regulator of UPR^mt^ (Nargund et al., 2012; Pellegrino et al., 2014). A similar transcriptional response has been described in mammals, identifying ATF5, which upon mitochondrial stress, fails to be imported into mitochondria for its degradation by mitochondrial protease lon protease homolog 1 but moves to the nucleus inducing gene transcription (**Figure 1**, UPR^mt^) (Fiorese et al., 2016).

Rimessi et al. demonstrated that CFTR deficiency reduced the mitophagic clearance during infection with detrimental repercussion on mitochondrial homeostasis, triggering an abnormal UPR^mt^ activation in CF airway epithelial cells (Rimessi et al., 2020b). An extensive nuclear ATF5 redistribution, associated to an increased expression levels of UPR^mt^ reporters, was measured in CF airway cells during *P. aeruginosa* infection. The persistent UPR^mt^ activation also increased the inflammatory-sensitivity of CF cells to pathogen, as shown by higher levels of NLRP3 inflammasome-dependent IL-1β and IL-18 released during infection (Rimessi et al., 2020b). The amplitude of inflammasome responses were correlated to amount of dysfunctional mitochondria accumulated and by the levels of nuclear ATF5. UPR^mt^ and NLRP3 inflammasome activation in turn led to worsening of mitophagic and xenophagic defects in CF cells, favoring a vicious cycle that contributed to exacerbate the *P. aeruginosa*-dependent cellular and mitochondrial stress (Rimessi et al., 2020b). These findings showed as the impairments in selective autophagy in CF are sustained by abnormal UPR^mt^ and NLRP3 activation, contributing to persistent damaged mitochondria and invading bacteria accumulation in CF cells.

## Apoptosis

During infection, in front of irreversible damages in response to prolonged stress conditions, mitochondria may activate the intrinsic apoptotic pathway (Green and Kroemer, 2004). Many pathogens activate the intrinsic apoptotic pathway, causing Δψ depolarization and mitochondrial permeabilization followed by enhanced levels of ROS and pro-apoptotic Bcl2 family proteins, such as Bax and Bid (Green and Kroemer, 2004; Wood et al., 2015). In turn, the excessive oxidative stress may oxidize mtDNA and active redox-sensitive kinases and transcription factors, which exacerbates the inflammatory environment contributing significantly to promote apoptosis in CF cells (**Figure 1**, apoptosis) (Jendrossek et al., 2001; Rottner et al., 2007; Kamdar et al., 2008; Galli et al., 2012; Rimessi et al., 2016).

Other studies suggest that the increased apoptotic susceptibility in CF cells is dependent by CFTR deficiency; e.g., I) due by abnormal intracellular Ca^2+^ signaling, which upon stress sensitizes the cell to organelle dysfunction and death (Antigny et al., 2011; Rimessi et al., 2020b); II) due to a reduced antioxidant activity, CFTR deficiency induced dysregulation of GSH concentration and transport, while reducing the expression of superoxide dismutase in F508del-CFTR mutant pancreatic cells (Gao et al., 1999; L’hoste et al., 2010; Kelly-Aubert et al., 2011; Rottner et al., 2011); III) due to a reduced expression of anti-apoptotic proteins, such as Bcl2 in CFTR-silencing intestinal cells^20^; IV) due to altered intracellular pH (de Meer et al., 1995; Barriere et al., 2001).

In any case, the enhanced apoptosis in CF cells contributes to hasten the tissue damage and functional loss in CF, worsening the disease state.

## Mito-Inflammation in Cystic Fibrosis

The extensive studies of these years revealed a supplementary role of mitochondria as drivers of inflammatory responses, leading us to reflect on new concept: the mito-inflammation. A compartmentalization of inflammatory process related to the role of mitochondria in engage and sustain the inflammatory responses and thus a druggable target to counteract the exacerbations of responses. In CF, mitochondria function as central regulator of danger signals, arbitrators with a double role in the pathogenesis of hyperinflammatory state.

Firstly, they act as checkpoints of intracellular downstream signal cascades to pathogen recognition receptor responses, induced by exogenous pathogen-associated molecular patterns. Bacterial ligands induce macrophage bactericidal-activity binding TLR 1, 2 and 4, which modulate the mitochondrial respiratory chain assembly factor, ECSIT, to increase mitochondrial ROS production (West et al., 2011). Again, the mitochondrial antiviral signaling protein (MAVS), located on the OMM regulates the transduction of interferon-dependent signaling pathway to amplify antiviral innate immune responses (Banoth and Cassel, 2018).

**Figure 2:**
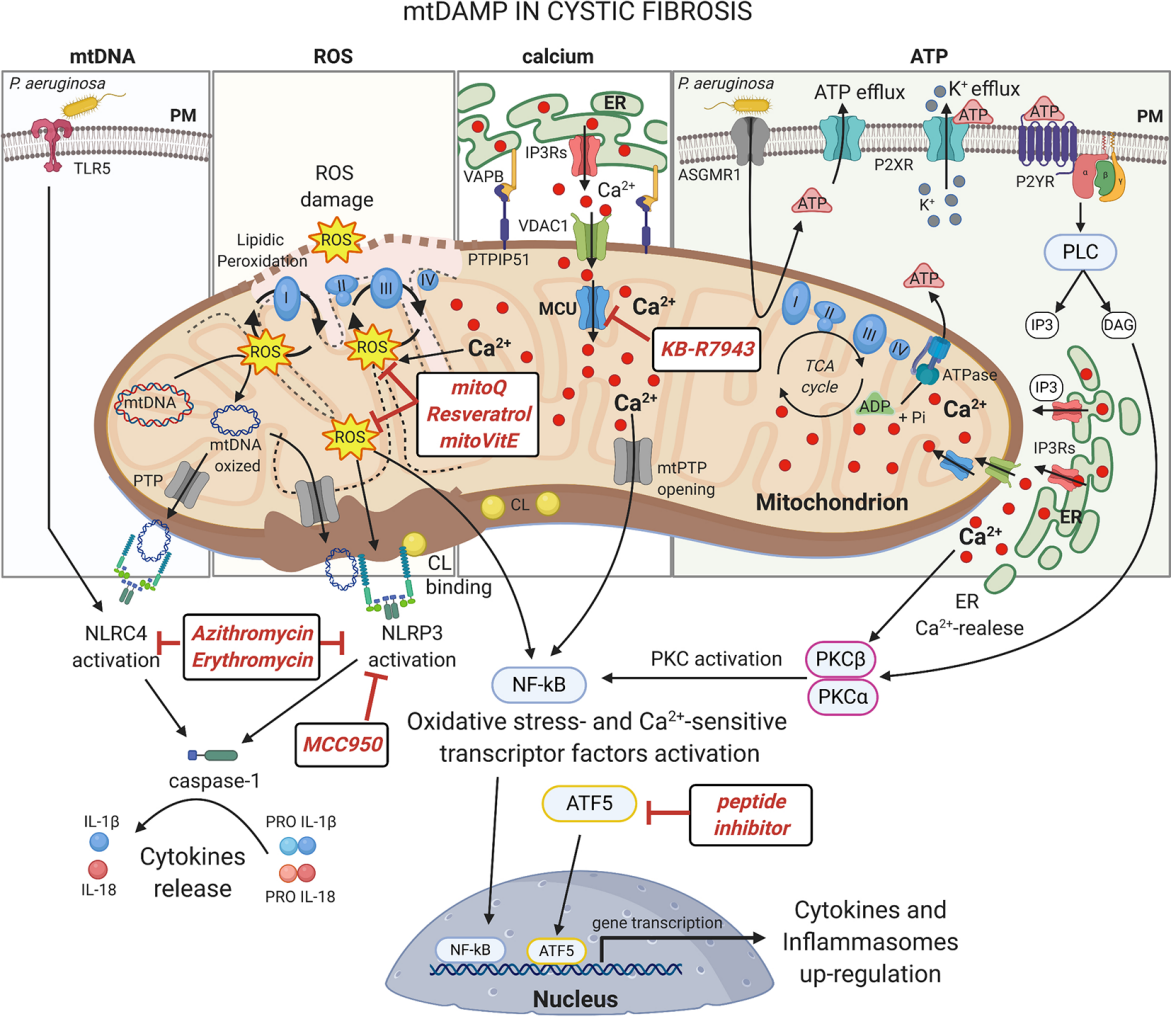
mtDAMPs in cystic fibrosis Under stress, mitochondria generate and/or release immunogenic molecules, named mitochondrial damage associated molecular patters (mtDAMPs), that influence significantly the inflammatory response in CF lung disease. The accumulation of dysfunctional mitochondria leads to excessive ROS production in CF airway cells with detrimental effects on mitochondrial constituents, inducing oxidative damages on mtDNA and mitochondrial respiratory complexes or the lipidic peroxidation of mitochondrial membranes. The ROS may also active: I) oxidative-stress sensitive transcriptor factors, which after nuclear translocation, up-regulate the expression of cytokines and inflammasomes; II) or the NLRP3 inflammasome to promote the release of interleukine-1β (IL-1β) and IL-18. NLRP3 and NLRC4 inflammasomes are also activated by direct interaction with mtDNA and cardiolipin (CL), released following mitochondrial damage or PTP opening, with consequent activation of caspase-1. The abnormal ER-mitochondria Ca^2+^ transfer, due by increased interorganelle crosstalk mediated by VAPB-PTPIP51 tethering during pathogen infection in CF, contributes to mitochondrial stress inducing ROS production, PTP opening and UPR^mt^ activation, which facilitate the activation of NF-kB and NLRP3 inflammasome. Increased levels of extracellular ATP, induced by *P. aeruginosa*-ASGMR1 signaling pathway, mediate ionic flux and intracellular potassium (K^+^) depletion through the ATP-binding to ligand-gated ion channels P2X receptors, sensitizing the CF airway cells to NLRP3 inflammasome activation. Indeed, mediating P2Y receptors, the extracellular ATP induces the Ca^2+^-dependent activation of classical Protein Kinase C (PKC) isoforms through the IP3-triggering ER Ca^2+^-release, resulting in NF-kB activation (Pinton et al., 2004). Several classes of molecules may exert anti-inflammatory activity targeting mitochondria at different levels. In this figure are shown the discussed drugs that may counteract the mito-inflammation in CF lung disease. Plasma membrane (PM); Toll-like receptor 5 (TLR5); NLR Family CARD Domain Containing 4 (NLRC4); permeability transition pore (PTP); NLR Family Pyrin Domain Containing 3 (NLRP3); Mitochondrial Calcium Uniporter (MCU); Voltage-dependent anion-selective channel 1 (VDAC1); Vesicle-associated membrane protein-associated protein B (VAPB); Protein tyrosine phosphatase interacting protein 51 (PTPIP51); Inositol 1,4,5-Triphosphate (IP3); diacylglycerol (DAG); Inositol trisphosphate receptors (IP3Rs); Endoplasmic Reticulum (ER); nuclear factor-κB (NF-kB); adenosine triphosphate (ATP); adenosine diphosphate (ADP); Asialo GM1 receptor (ASGMR1); P2X purinoceptor (P2XR); Purinergic Receptor (P2YR); Phospholipase C (PLC). This figure has been created with “BioRender.com”.

Second, mitochondria act as a key source of mitochondrial danger-associated molecular patterns (DAMPs), where ROS, mtDNA, ATP, CL and Ca^2+^ are released as danger signals into the cytosol or in the extracellular milieu, which recognized by TLRs and/or the cytosolic nucleotide-binding oligomerization domain-like receptors (NLRs) trigger the inflammatory responses inducing the expression and secretion of numerous pro-inflammatory mediators (**Figure 2**) (Lavelle et al., 2010).

Upon stress, the mitochondrial ROS production results excessive in CF cells, determining (**Figure 2**, ROS): I) oxidative damage to intracellular macromolecules, including mtDNA; II) activation of redox-sensitive transcription factors, inducing the up-regulation of cytokines and inflammasomes. The transcription factors NF-kB, AP-1 and HIF-1 result hyperactivated in murine and human CF airway cells, due to also by intrinsic defects associated to defective CFTR (like abnormal intracellular Ca^2+^ signaling), causing production of chemokine IL-8 and priming of NLRP3 and pro-IL-1β expression (McNamara et al., 2006; Tabary et al., 2006; Levy et al., 2009; Legendre et al., 2011; Saadane et al., 2011; Nichols and Chmiel, 2015); III) NLRP3 inflammasome activation; and IV) additional mitochondrial impairments in a feed-back stimulatory manner that exacerbates the inflammatory response (Brookes et al., 2004; Rimessi et al., 2016).

mtDNA may be oxidized by ROS and its damages may alter the OXPHOS activity, producing additional ROS (**Figure 2**, mtDNA). mtDNA may escape from matrix, due to impaired mitophagy or Ca^2+^- and oxidative stress-dependent mPTP opening, and binds directly the NLR component of inflammasomes to trigger theirs activation (Escames et al., 2012). CL is accidentally released from damaged mitochondria and acts as direct activator of NLRP3 inflammasome in the cytosol (Iyer et al., 2013).

ATP released from mitochondria acts in the extracellular milieu as ligand of purinergic receptors, resulting in increased ROS production and cytokines release, influencing also NLRP3 activation (Bonora et al., 2012). Finally, dysfunctional mitochondria present altered mitochondrial Ca^2+^ signals, critical for inflammation, which influence the mitochondrial stress responses with consequent repercussions on inflammasome activation and on up-regulation and release of cytokines (Rimessi et al., 2015).

The mitochondrial DAMPs are preferentially bound by different NLRs to modulate the amplitude of immune responses activating the inflammasomes, multiprotein complexes that lead to caspase-1 activation and subsequent maturation of the pro-inflammatory cytokines IL-1β, IL-18, and IL-33 (**Figure 2**) (Broz and Dixit, 2016). Elevated concentrations of IL-1β and IL-18 were detected in human airway epithelial cells, monocytes and serum of CF patients (Levy et al., 2009; Rimessi et al., 2015; Iannitti et al., 2016; Scambler et al., 2019). Mitochondrial DAMPs are the principal initiator of NLRP3 inflammasome activation. NLRP3 is a cytosolic receptor that, once activated, interacts with mitochondria where oligomerizes recruiting the adaptor protein apoptosis-associated speck-like protein containing a caspase recruitment domain (ASC) and the procaspase-1 (Zhou et al., 2011). Its mitochondrial localization and association depends by MAVS, which promotes also its activation, and by mitochondrial-anchored protein ligase (Park et al., 2013; Subramanian et al., 2013). Rimessi et al. showed the NLRP3 mitochondrial recruitment and activation in CF airway cells during *P. aeruginosa* infection, since defective CFTR induced mitochondrial Ca^2+^-overload and ROS production, which in turn driven the NLRP3 inflammasome activation and release of IL-1β and IL-18 (Rimessi et al., 2015). The increased susceptibility to pathogen-dependent mitochondrial dysfunction and the mitochondrial Ca^2+^-overload in human CF airway cells resulted in the recruitment of both NLRP3 and NLRC4 inflammasome with consequent worsen of inflammation (Rimessi et al., 2015). NLRC4/IPAF inflammasome activation has been associated to mitochondrial DAMPs during *P. aeruginosa* infection also in macrophages, due to ROS and direct binding with oxidized mtDNA (Jabir et al., 2015). Typically, it activated by TLR5-recognition on PM *via* bacterial flagellin and in the cytosol *via* the microbial type III secretion system (Sutterwala et al., 2007; Tolle et al., 2015). The interplay between NLRP3 and NLRC4 inflammasome has been also reported in CFTR-null mouse model and in alveolar CF macrophages and neutrophils, where NLRP3 greatly contributed (Iannitti et al., 2016). In CF airway cells, NLRP3 inflammasome activation induces also the downregulation of mitophagy and xenophagy, promoting the accumulation of damaged mitochondria and invading bacteria into the cells (Rimessi et al., 2020b). In turn, released IL-1β induces mitochondrial ROS production, down-regulating the complex-I activity and Δψ, and NF-kB activation, generating a loop that sustain and exacerbate the inflammatory response (Lopez-Armada et al., 2006; Escames et al., 2012).

The hyperinflammation observed in CF lung is in part conditioned by altered phenotype of airway immune cells associated to defective CFTR. CF neutrophils exhibit altered chlorination of phagocytosed bacteria while the macrophages showed reduced selective autophagic activity, becoming a replicative niche for bacteria (Painter et al., 2006; Lamothe and Valvano, 2008; Ratner and Mueller, 2012; Assani et al., 2014). Mitochondrial metabolism and the metabolic state of the cell drive the pro- or anti-inflammatory responses, regulating the polarization and activation of different immune cells, including neutrophils and macrophages (Michalek et al., 2011; Tarique et al., 2015). Accordingly, a compromised metabolism was found in CF neutrophils in response to LPS, shifting to a state of increased aerobic glycolysis with consequent exacerbation of IL-1β production (McElvaney et al., 2019). CF macrophages did not respond to IL-13/IL-4 and failed to polarize into M2, contributing to excessive production of cytokines (Tarique et al., 2017). The pro-inflammatory Th17 cells are highly glycolytic; meanwhile the immunosuppressive Treg cells present an elevated rate of lipid oxidation. In CF patients, the differentiation of T lymphocytes to Th17 phenotype was increased (Kushwah et al., 2013), indicating that the maintenance of mitochondrial homeostasis and of a functional metabolic reprogramming are critical conditions to regulate the immune responses also in CF.

## Targeting Mito-Inflammation as Therapeutic Approach in Cystic Fibrosis

In CF, the ideal drug should be one that interrupts the vicious CF cycle that sustains lung hyperinflammation, but also generates favorable conditions to rescue or potentiate the residue functionality of defective CFTR. In this regard, several modulators of mitochondrial behaviors have shown anti-inflammatory activity, such as mitochondrial antioxidants, modulators of mitochondrial Ca^2+^-exchange, selective autophagic-inducing compounds and inflammasome and IL-1β inhibitors.

Strategies that limit mitochondrial ROS production may be useful to control the hyperactivation of redox-sensitive inflammatory transcription factors and inflammasomes (**Figure 2**, ROS). An example may be found in the natural phenolic antioxidant resveratrol (3,4′,5-trihydroxystilbene), which efficiently reduces oxidative stress and subsequent inflammation, preserving mitochondrial Δψ and mtDNA *in vitro* and *in vivo* (Manna et al., 2000; Xu et al., 2012). At high concentration, in preclinical studies resveratrol rescues the expression of F508del-CFTR, the chloride secretion and the intracellular transport in human primary airway epithelial cells and CF mouse models (Hamdaoui et al., 2011; Dhooghe et al., 2015; Lu et al., 2020). Indeed, in presence of the modulator, ivacaftor, resveratrol augmented the G155D-CFTR activation in human primary sinonasal cells (Cho et al., 2019). Unfortunately, resveratrol exhibits poor bioavailability with maximal achievable plasma concentration of about 2 μM (a concentration no effective to improve CFTR function), that could limit its clinical usefulness (Walle, 2011; Jai et al., 2015). However, different therapies in CF involve topical application of drugs to lung mediating aerosol, a strategy that may overcome the limit of bioavailability.

Other antioxidants, such NAC, have been locally administrated to CF adults and children in different clinical trials. Acute administration of NAC was found to be well tolerated and free of adverse effects. Despite this, it was only found beneficial variations in sputum rheology and hydration, two factors that may be predictive of improved airway mucus clearance. Any significant change in pulmonary function and in clinical indicator were registered (App et al., 2002). Of more interest were the results obtained in a phase II randomized placebo-controlled trial, where it was performed long-term treatment with oral NAC (Conrad et al., 2015). Indeed, authors found improvements in lung functions, reduction in the incidence of pulmonary exacerbations. Unfortunately, they found no change in sputum human neutrophil elastase activity and other biomarkers of inflammation, suggesting to consider more specialized antioxidants as therapeutic strategy in CF (Cantin, 2004). Antioxidants that have shown promising results in oxidative stress-related diseases are mitovitamin E (MitoVit-E) and mitoquinone (Mito-Q), mitochondrial antioxidants that contain the triphenylphosphonium cation moiety that facilitates theirs accumulation into the organelle (Smith et al., 2003). MitoVit-E suppressed the sepsi-induced peripheral and myocardial production of cytokines, including IL-1β, improving mitochondrial function and heart activity, while Mito-Q restored mitochondrial function in chronic obstructive pulmonary disease (COPD) patients, reducing inflammation and IL-8 release in preclinical studies (Zang et al., 2012; Wiegman et al., 2015). In CF patients, the low level of vitamin-E in serum is normally correct with a dietary supplement, this implementation should be enriched also with MitoVit-E to safeguard the mitochondrial function (Sokol et al., 1989).

The hyperinflammation in CF lung is due also by altered mitochondrial Ca^2+^ exchange, in which MCU is involved (Rimessi et al., 2015; Rimessi et al., 2020b). Pharmacological MCU inhibition, mediating KB-R7943, attenuated *in vitro* and *in vivo* the *P. aeruginosa*-dependent mitochondrial dysfunction and hyperinflammation in CF lung, controlling UPR^mt^ and NLRP3 inflammasome activation (**Figure 2**, calcium) (Rimessi et al., 2020b). KB-R7943 is the first freely PM permeable and only one available dose-selective MCU inhibitor, in opposing to more used MCU inhibitors, Ruthenium Red and Ru360 that are cell impermeable (Santo-Domingo et al., 2007). A new class of cell-permeable selective MCU inhibitors are now available, and Ru265 and DS16570511 are minimally toxic *in vitro* and could have important implications in CF in the future (Kon et al., 2017; Woods et al., 2019). KB-R7943 also restored mitophagy and xenophagy in CF, removing stressed mitochondria and pathogens able to induce mtDAMPs release, it attenuated the inflammatory state (Rimessi et al., 2020b). Thus, selective autophagic inducers may mitigate the hyperinflammation in CF. Rapamycin and MK-2206, both inhibitors of PI3K/Akt/mTOR pathway, reduced the severity of CF inflammation and improved the CFTR stability in airway epithelial cells in preclinical studies (Abdulrahman et al., 2011; Reilly et al., 2017). In a perspective of mitochondrial quality control–based therapy, the synthetic cell-penetrating dominant negative ATF5 peptide (Cs Bio^®^) should be useful to dampen the persistent UPR^mt^ activation in CF, restoring the autophagy process (**Figure 2**) (Sheng et al., 2011).

Inflammasome and IL-1β inhibitors may rescue selective autophagy and attenuate the hyperinflammation in CF. MCC950 inhibits NLRP3 and AIM2 inflammasome, but not NLRC4, blocking the formation of ASC complexes (Coll et al., 2011). *In vivo*, MCC950 improved the clearance of *P. aeruginosa* and reduced IL-1β release in the lung (McElvaney et al., 2019). The pathogenic NLRP3 activity in CF may be reduced using the IL-1 antagonist receptor Anakinra, that by blocking the biological IL-1α and IL-1β activity ameliorated tissue damage and inflammation against *P. aeruginosa* in CF, with positive repercussion also on autophagy and neutrophils infiltration (Iannitti et al., 2016). The questionable limit of this approach is the indiscriminate block of IL-1 activity, which may compromise the global inflammatory responses important in fighting the persistent infections, in particular in those body areas where the microbe-host interactions are strategic. Despite this consideration, a phase 2 study (ClinicalTrials.gov Identifier: NCT03925194) to evaluate safety and efficacy on lung function of Anakinra subcutaneous administration in CF patients is ongoing. Recently, it has been demonstrated that also azithromycin and erythromycin (macrolides used in antibiotic CF therapies) inhibit the activation of inflammasome NLRP3 and NLRC4, which attenuated lung injury and inflammation enhancing the *P. aeruginosa* clearance in mice and in bronchiectasis patients (**Figure 2**) (Fan et al., 2017). Considering that the beneficial effects of these antibiotics is difficult to maintain over the years, treatments engaging specific inflammasome inhibitors may be used to improve the effects of these drugs in long-term treatment (Samson et al., 2016).

In conclusion, all these studies reveal the pathogenic role of mito-inflammation in CF and support mitochondria as new pharmacological targets. Emerging concepts of mitochondrial quality control provide opportunities to develop mitochondrial therapies, which aim to preserve the mitochondrial function as an alternative anti-inflammatory approach. The therapeutically restoring of mitochondrial homeostasis will be useful to improve the clinical state of CF lung disease, avoiding the overstimulation of inflammatory signals.

## Author Contributions

All authors contributed to the article and approved the submitted version.

## Funding

The research was funded by grants from Italian Cystic Fibrosis Research Foundation FFC#12/2010 and #19/2014, Italian Association for Cancer Research (AIRC, IG-23670), Telethon (GGP11139B), local funds from the University of Ferrara, and the Italian Ministry of Education, University and Research (PRIN grant 2017E5L5P3) to PP. SP is supported by Fondazione Umberto Veronesi. AR is supported by Italian Cystic Fibrosis Research Foundation grant FFC #20/2015, local funds from University of Ferrara, FIR-2017, the Italian Ministry of Health (GR-2016-02364602), and the Italian Ministry of Education, University and Research (PRIN grant 2017XA5J5N). PP is grateful to Camilla degli Scrovegni for continuous support.

## Conflict of Interest

The authors declare that the research was conducted in the absence of any commercial or financial relationships that could be construed as a potential conflict of interest.
